# In Vitro and In Vivo Models of Non-Alcoholic Fatty Liver Disease: A Critical Appraisal

**DOI:** 10.3390/jcm10010036

**Published:** 2020-12-24

**Authors:** Pierre-Antoine Soret, Julie Magusto, Chantal Housset, Jérémie Gautheron

**Affiliations:** 1 Centre de Recherche Saint-Antoine (CRSA), Sorbonne Université, Inserm, 75012 Paris, France; pasoret@hotmail.com (P.-A.S.); julie.magusto@inserm.fr (J.M.); chantal.housset@inserm.fr (C.H.); 2 Assistance Publique-Hôpitaux de Paris (AP-HP), Hepatology Department, Reference Center for Inflammatory Biliary Diseases and Autoimmune Hepatitis, Saint-Antoine Hospital, 75012 Paris, France; 3 Institute of Cardiometabolism and Nutrition (ICAN), Sorbonne Université, Inserm, AP-HP, 75013 Paris, France

**Keywords:** NASH, NAFLD, mouse model, spheroids, organoids, liver-on-a-chip, cell culture

## Abstract

Non-alcoholic fatty liver disease (NAFLD), including non-alcoholic fatty liver (NAFL) and non-alcoholic steatohepatitis (NASH), represents the hepatic manifestation of obesity and metabolic syndrome. Due to the spread of the obesity epidemic, NAFLD is becoming the most common chronic liver disease and one of the principal indications for liver transplantation. However, no pharmacological treatment is currently approved to prevent the outbreak of NASH, which leads to fibrosis and cirrhosis. Preclinical research is required to improve our knowledge of NAFLD physiopathology and to identify new therapeutic targets. In the present review, we summarize advances in NAFLD preclinical models from cellular models, including new bioengineered platforms, to in vivo models, with a particular focus on genetic and dietary mouse models. We aim to discuss the advantages and limits of these different models.

## 1. Introduction

Non-alcoholic fatty liver disease (NAFLD) comprises two histological forms: non-alcoholic fatty liver (NAFL) and non-alcoholic steatohepatitis (NASH) [1]. NAFL is defined as the presence of hepatic steatosis without hepatocellular necrosis and no or minimal inflammation, while NASH is characterized by the association of steatosis, liver inflammation and hepatocyte ballooning, with or without fibrosis. The progression of inflammation and fibrosis in NASH underlies cirrhosis and hepatocellular carcinoma (HCC) [1]. NAFLD is the hepatic manifestation of obesity and metabolic syndrome, affecting up to one-third of the adult population in the western world [2,3]. Thus, NAFLD is rapidly becoming a worldwide public health problem with an exponential growth prediction within the 10 next years due to high caloric intake combined with a sedentary lifestyle [4]. NASH is also predicted to become the most common indication of liver transplantation in the near future [5,6]. Molecular mechanisms leading to NASH and its evolution to liver fibrosis and cirrhosis remain partially unknown. A “two-hit” hypothesis has been postulated [7]. The first hit is defined as fat accumulation in the liver, which triggers an inflammatory process that results in steatohepatitis and fibrosis. The nature of the second hit is not fully understood, but it may involve oxidation of fatty acids and subsequent free-radical-induced liver damage. Although widely accepted, this theory is now questioned, as dysfunction in metabolic pathways such as lipid transport and insulin signaling, as well as gut microbiota and genetic polymorphisms, are important pathogenic drivers contributing to disease heterogeneity [7,8]. Even if NAFLD is the most common chronic liver disease, no pharmacological treatment is currently approved for this disease. Therefore, basic and preclinical research remains essential to improve our understanding of NAFLD pathophysiology and for the development of pharmacotherapies. The biological systems used in this research range in complexity and scale from monolayer cell culture to complex three-dimensional (3D) organoids and model organisms. Here, we review advances in the cellular and in vivo models currently used to study NAFLD.

## 2. Materials and Methods

We used NCBI PubMed and ScienceDirect databases for literature research. Publications in English were searched using the terms: NAFLD, NASH, liver steatosis, mouse models of NAFLD, non-rodent models of NAFLD, in vitro cell culture models of NAFLD, spheroids, organoids, liver-on-a-chip. Articles were screened to identify potentially relevant studies. Reference lists of retrieved literature were also screened.

## 3. In Vitro Cell Culture Models of Non-Alcoholic Fatty Liver Disease

### 3.1. Hepatic Cell Sources

**Figure 1:**
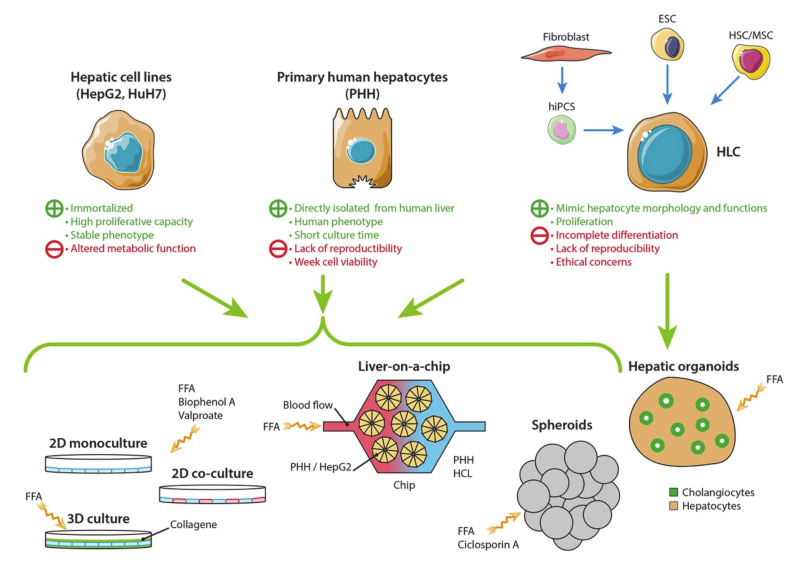
Hepatic cell sources and in vitro models of non-alcoholic fatty liver disease (NAFLD). Different cell sources are now available to build in vitro models of NAFLD. Primary human hepatocytes (PHH) display the closest phenotype to the human liver. A short culture time and low availability of PHHs are alleviated by using immortalized cell lines (HepG2, HuH7 or HepaRG) and hepatocyte-like cells (HLC) derived from stem cells (as human-induced pluripotent cell—hIPSC, embryonic stem cells—ESC or hepatic stem cells—HSC, or mesenchymal stem cells—MSC). However, altered metabolic functions and incomplete differentiation of HLC are limitations of the model. Several models have been developed to mimic NALFD in vitro. NAFLD can be studied in 2- or 3-dimensional cell cultures by adding free fat acid (FFA), Bisphenol A or Valproate in the culture medium. New 3D models have been recently developed: spheroids are derived from different hepatic cell sources; hepatic organoids are produced by stem cell differentiation in parenchymal and non-parenchymal liver cells. Livers-on-a-chip are devices designed to mimic the physiological environment of the liver lobule: hepatocytes are placed inside a micro-scaffold, and a fluidic flow passes through the chip to reproduce blood circulation.

Cell lines are widely used in research and drug development. They display a higher replicative capacity than primary cells and a stable phenotype so that they can be used for a long period-of-time. They are also inexpensive. Primary cells are directly issued from tissues. In contrast to cell lines, they have a limited life and expansion capacity, but they maintain the morphological and functional characteristics of their origin for a short period of time. Here, we review the cell culture models and discuss their advantages and limitations to study the development and progression of NAFLD (Figure 1).

#### 3.1.1. Human Hepatic Cell Lines

Human cell lines are immortalized cells obtained from resected tumor tissue (e.g., HepG2, HepaRG or HuH7 hepatoma cell lines) or generated by the genetic manipulation of primary liver cells (e.g., SV40 Large T, hTERT) [9,10,11]. The principal advantages of immortalized cell lines are their unlimited growth potential and a stable phenotype throughout culture time [12]. Thus, using these models facilitates standardized protocols and reproducible studies. However, due to their tumor phenotype or the immortalization process, hepatic cell lines are generally altered in certain metabolic functions that limit a direct comparison to the human situation, especially when studying NAFLD. The available immortalized human hepatic cell lines and their in vitro application other than NAFLD have been reviewed here [13,14].

#### 3.1.2. Primary Human Hepatocytes

Primary human hepatocytes (PHHs) are cells isolated from resected liver tissue using a two-step collagenase perfusion or magnetic cell separation technique and maintained in culture for a few days [15,16]. Due to direct isolation from tissue, PHHs are the closest model to mimic the phenotype of a hepatocyte in vivo; notably, their plasma membrane maintains active uptake/secretion mechanisms and metabolism [9,17]. However, PHHs show phenotypic instability, and their accessibility and culture time are limited [18,19]. Moreover, human sample collection requires ethical authorization, and non-pathological tissues are necessary and difficult to obtain in clinical practice. Cell viability is highly dependent on the surgical and transport conditions of tissue collection, and genetic variability between donors can introduce a bias leading to a lack of reproducibility between studies and experiments [20,21]. Therefore, PHHs appear particularly suitable for drug metabolism studies that do not require a long period of culture time, and to a lesser extent for modeling specific liver diseases such as NAFLD [17].

#### 3.1.3. Hepatocyte-Like Cells

Hepatocyte-like cells (HLCs) are cells differentiated from human stem cells (hSCs), including liver stem cells and embryonic pluripotent stem cells [22]. Human SCs can be differentiated into HLCs in vitro using combined exposure to growth factors and nutrients [23,24]. Furthermore, techniques now exist for the production of human-induced pluripotent stem cells (hiPSCs) from somatic cells such as fibroblastic cells, which represent an unlimited source of stem cells that can secondarily differentiate into HLCs [25,26,27]. HLCs display morphological and functional characteristics that are similar to those of primary hepatocytes but without culture time limitation [28]. Nevertheless, HLCs also raise issues regarding the ethical use of embryonic stem cells, the incomplete hepatic differentiation and the lack of standardized differentiation protocol, which limits studies reproducibility [20].

### 3.2. Two-Dimensional Cell Culture Models

#### 3.2.1. Monoculture

The most commonly used type of cell culture is the two-dimensional (2D) model. To mimic NAFLD in 2D cultures of hepatocytes of different sources as outlined in Section 3.1, steatosis can be induced by adding free fatty acids (FFA) to the cell culture medium, especially oleic and/or palmitic acids [15,29]. The induction of hepatocytes with FFA leads to triglyceride accumulation in their cytoplasm, which ultimately causes endoplasmic reticulum (ER) stress, inflammation and cell death, which are all key hallmarks of NASH [30,31]. Steatosis can also be induced in cultured cells using drugs such as Bisphenol A, which increases lipid accumulation due to SREBP1 upregulation, or Valproate, which enhances fatty acid uptake and triglyceride synthesis [32,33]. Additionally, triglyceride accumulation can be enhanced in the presence of an inducer of ER-stress [34]. Hepatocyte 2D monoculture allows the study of all major metabolic pathways, namely carbohydrates, lipids and amino acids [11,35,36]. It is also suitable for preclinical drug testing to evaluate drug efficacy and cellular tolerance [17,37]. However, 2D human monocellular models fall short of expectations due to the absence of critical hepatocyte–non-parenchymal-cell (NPC) interactions, which are important for the initiation of inflammatory processes leading to fibrogenesis in NAFLD. 

#### 3.2.2. Co-Culture

2D co-culture models are used to study the mutual interaction between hepatocytes and NPCs in vitro. Different cell types are grown together in the same environment, and steatosis can be induced like in monoculture by adding FFA to the culture medium. One model of particular interest is the co-culture of hepatocytes with hepatic stellate cells (HSCs) that are key effectors of fibrosis in NAFLD [38]. This interplay has been studied by Barbero-Becerra et al., who developed a model of co-culture of HuH7 (human hepatocyte cell line) and LX-2 (Human HSC line) cells [39]. They demonstrated that FFA exposure induced the expression of α-SMA in LX-2 cells only when they were simultaneously co-cultured with Huh-7 and that HSC activation was independent of FFA accumulation but required cell-to-cell interaction with hepatocytes. Co-culture of primary hepatocytes with Kupffer- or endothelial-cells provides a powerful tool to assess the mechanisms whereby FFA triggers inflammatory processes in vitro [40,41]. However, co-culture is quite difficult to implement, as, for example, growth medium optimization is required to select the medium that best sustains the different cell populations. Human cells are also poorly available, adding another difficulty to the co-culture models [42,43]. For all these reasons, these models are rare in the literature compared to monoculture and animal models.

### 3.3. Three-Dimensional Cell Culture Models

In the past decade, the development of three-dimensional (3D) culture models has dramatically increased the knowledge of cellular and molecular mechanisms in the pathogenesis of liver diseases, especially NAFLD, and offers new opportunities for drug testing in this context.

#### 3.3.1. 3D Cell Culture in a Collagen Gel Sandwich

In this model, hepatocytes are placed between two layers of collagen gel, allowing them to reconstruct the cellular polarity normally found in the liver. In this configuration, sandwiched hepatocytes maintain the secretion of albumin, transferrin, fibrinogen, bile acids and urea for at least 6 weeks, whereas hepatocytes cultured on a single layer of collagen gel cease such secretion between 1 and 2 weeks [44]. This secretion can be restored when a second layer of collagen is applied to the monolayer of collagen gel culture [44]. Re-establishment of bi-polarity allows one to prolong survival and to maintain hepatic metabolism in PHHs [19,45]. Recently, a more complex 3D model has been developed where hepatocytes are co-cultivated with liver sinusoidal endothelial cells (LSECs) inside collagen gels to mimic liver lobular architecture [40]. Hepatocytes and LSECs account for approximately 80% of the liver mass where the LSECs line the sinusoidal walls, and act as a barrier between hepatocytes and blood. This co-culture configuration provides an environment wherein hepatocyte and LSECs, through cell–cell contacts and/or soluble factors, lead to enhanced cell function and survival [40]. However, cell–cell interactions can be hidden or blocked due to collagen thickness in this model [46]. Thus, the sandwich configuration has been rarely used as an NAFLD model. 

#### 3.3.2. Hepatic Spheroids and Organoids

Spheroids or 3D cell aggregates are now the most commonly used 3D culture model. Hepatic spheroids are aggregates of hepatocytes usually derived from liver progenitor cells but also consisting of PHHs. The spheroid formation is initiated by spontaneous self-aggregation of the hepatocytes and does not require extracellular matrix to develop. Several techniques have been developed based on non-adherent surfaces and gravitational adherence [47,48]. PHHs in spheroids remain differentiated for at least 3 to 4 weeks, and hepatic steatosis can be induced by supplementing the culture medium with pathophysiological concentrations of FFA, carbohydrates and insulin [49,50]. In another model of PHH spheroids, hepatic steatosis can be induced by Cyclosporine A [51]. Hepatic spheroids can be built together with NPCs such as stellate cells and endothelial cells to develop more complex liver-like structures [50,52,53,54]. However, in standard non-adhesive plates, it is difficult to maintain uniform spheroid size, and clusters of spheroids can emerge that limit nutrient and oxygen absorption to the distal spheroids, leading to cell mortality [55].

Organoids are new research tools, which are defined as artificially grown masses of cells that resemble miniature organs. Liver organoids are obtained through isolation and expansion of stem and progenitor cells from hepatic stem cell niches to form small self-organizing 3D structures that simulate many of the functions of a native liver [56,57]. Successful organoid formation requires a careful orchestration of spatiotemporal signals from growth factors to supportive matrices in order to stimulate the different cell niches. To create human liver organoids (HLOs), human pluripotent stem cells are co-differentiated into epithelial and mesenchymal lineages to form spheroids. These spheroids are then embedded in Matrigel and cultured with retinoic acid, and hepatocyte differentiation is achieved using a specific maturation medium. Ouchi et al. have shown that the resulting HLOs are self-organized clusters of cells comprising liver epithelial cells (cholangiocytes and hepatocytes) and NPC (stellate-, biliary stem- and Kupffer-cells) [58]. When these HLOs are exposed to FFA, hepatocytes developed steatosis and ballooning, Kupffer cells released pro-inflammatory cytokines, and stellate cells produced collagen, three key processes in NASH [59]. In addition, increased ROS levels and overexpressed lipid- and carbohydrate-related genes were observed [60]. More recently, Collin de l’Hortet et al. developed HLOs based on the control of the expression of SIRT1 (silent information regulator 1), known to exert protective functions in hepatocytes and macrophages by modulating metabolic and inflammatory pathways, respectively [61,62]. In these HLOs, the downregulation of SIRT1 induces a rapid accumulation of lipid droplets in hepatocytes accompanied by a pro-inflammatory response of the neighboring cells. Moreover, the majority of the metabolic pathways seen in livers of NAFLD patients were also upregulated in these HLOs [61,62]. Thus, HLOs appear to be promising tools for pathophysiological studies in NAFLD. As compared to 2D cell culture, the 3D nature of organoids can more closely mimic natural physiological processes, including stem cell differentiation, cellular movement and cell–cell interactions. In addition, organoids can undergo extensive expansion and culture and maintain their genomics stability, making long-term storage and high-throughput screening possible [63]. As compared with animal models, organoids reduce animal experiments and are easily accessible to live imaging techniques, and in some instances, they can provide a more accurate model of human development and disease than animal models do [63]. However, cell differentiation remains sometimes incomplete, and cell organization is random, which leads to a lack of reproducibility. Methods and techniques still need further improvement to reach a complete differentiation and a standardized architecture of the organoids. 

#### 3.3.3. Liver-On-A-Chip Technology

Liver-on-a-chip is based on microstructures and microfluidic devices aimed to establish microscale functional liver constructs on a chip. Basically, the microarchitecture of the liver is mimicked by a polymeric scaffold that consists of hundreds of small channels [64]. The dimensions are such that when hepatocytes are seeded into these channels, they form a long donut-shaped arrangement that closely resembles the hepatic lobule that a microfluid containing various nutrients and oxygen can pass through to simulate blood flow [65]. A more complex system can be engineered by integrating NPCs in a vascular layer comprising endothelial cells and macrophages to a hepatic layer comprising stellate cells co-cultured with hepatocytes [66]. The 3D culture is then embedded in a microfluidically perfused biochip that enables sufficient nutrition supply and reproduces the morphological aspects of the human liver sinusoid [66]. In this model, the perfusion chamber provides a fluidic shear stress and mimics the microenvironment of native liver [66].

Liver-on-a-chip has been extensively studied to create in vitro NAFLD models. Gori et al. have cultured HepG2 cells under free fatty acid (FFA) supplementation in a microfluidically perfused device, which mimics the endothelial–parenchymal interface of a liver sinusoid and allows the diffusion of nutrients and removal of waste products similar to that in liver microcirculation [67]. In this chip, the microfluidic dynamic allows gradual and lower intracellular lipid accumulation, higher hepatic cell viability and minimal oxidative stress compared with static cultures. It closely recapitulates the chronic conditions linked to the fat accumulation that occurs in NAFLD patients [67]. Likewise, Kostrzewski et al. cultured PHHs in a 3D perfused platform and showed that fat accumulation in the cultured cells was gradual over a long period of time [59]. Moreover, the metabolic activities (e.g., cytochrome P activity) of PHHs were reduced gradually across culture time in line with what has been observed in the liver of NAFLD patients [59]. Lee et al. also built a “gut-liver-on-a-chip” to study the gut–liver axis in the context of NAFLD [68]. In this microfluidic chip, FFAs were absorbed through a gut layer, and the subsequent secretion of chylomicrons was observed and coincided with fat accumulation in hepatocytes.

Liver-on-a-chip models dramatically increase the possibility to model NAFLD in vitro, including interplay between liver and other organs, which is an essential way to understand metabolic disorders. Compared to organoids, the chip technology is more reproducible because of standardized protocols and bioengineering fabrication. However, due to high complexity and cost, it is not widely spread in laboratories.

### 3.4. Human Precision-Cut Liver Slices Model

Human precision-cut liver slices (hPCLS) represent a robust ex vivo model in which multi-cellular histoarchitecture of the hepatic environment remains functional for at least 5 to 21 days in culture, although viability usually decreases afterward [69,70,71,72]. Inflammation is a key feature to distinguish steatohepatitis from simple steatosis. Thus, hPCLS that retain liver-infiltrating immune cells such as lymphocytes and macrophages allow one to study the changes observed during the different stages of NAFLD and to test the therapeutic efficacy of new compounds [71,73,74]. Hepatic steatosis can be induced by adding FFA to the culture medium of hPCLS, which results in the activation of inflammation and fibrogenesis in NPCs [71,75]. This model is also well suited to understand whether intrahepatic lipid accumulation can be modulated through epigenetic manipulation [71].

Taken together, co-culture or 3D models that mimic the architecture of liver tissue are powerful tools to elucidate the cellular mechanisms of fatty liver disease in order to study cell interactions in the progression of the disease from simple steatosis to steatohepatitis. However, further research is needed to standardize the culture conditions and to increase the availability of last-generation technologies.

## 4. Mouse Models of Non-Alcoholic Fatty Liver Disease

### 4.1. Dietary Mouse Models of NAFLD

#### 4.1.1. High-Fat Diet Feeding

**Figure 2:**
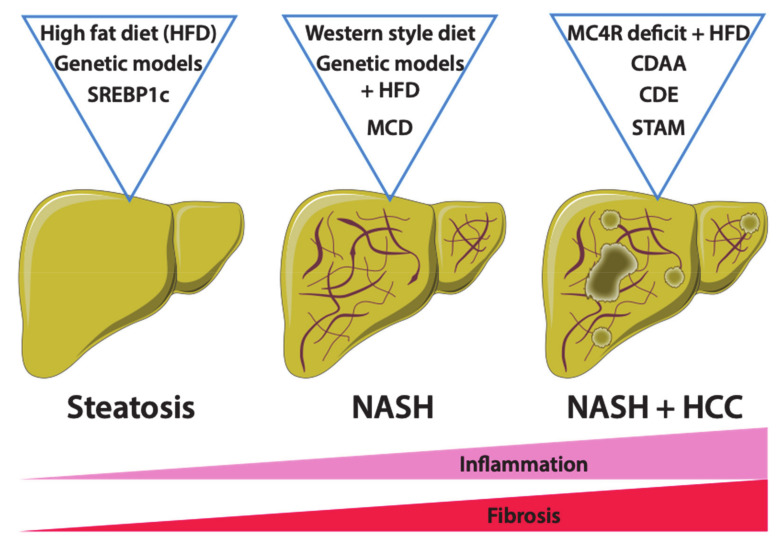
Mouse models of NAFLD. Several mouse models have been described to mimic NAFLD in vivo. Both genetic and diet models are widely used to create experimental conditions of NAFLD and for preclinical drug testing. However, each model is specific as mice do no present all the typical features of NAFLD, from non-alcoholic fatty liver (NAFL) and non-alcoholic steatohepatitis (NASH) and hepatocellular carcinoma (HCC). High-fat diet (HFD) and Western diet-fed mice, as well as genetic ob/ob (leptin deficiency) and db/db (leptin receptor mutation) mouse models, display metabolic syndrome and severe steatosis but no liver inflammation or fibrosis. However, the Methionine- and Choline-deficient diet (MCD) and its derivate diets such as Choline Deficient Ethionine-supplemented (CDE) and Choline-deficient L-amino acid-defined (CDAA) diets promote liver inflammation and fibrosis. The stelic animal model (STAM) associates a toxic injection of streptozotocin with an HFD, resulting in HCC development. Other genetic models, such as Melanocortin-4 receptor (MC4R) deficiency or *Sterol regulatory element-binding proteins (SREBP1c)* overexpression, predispose to metabolic syndrome and obesity but need a second hit (such as HFD) to trigger NASH.

Many studies have demonstrated that a diet rich in fat represents a major risk factor for the development of obesity and associated metabolic disorders such as NAFLD [76]. Accordingly, diets rich in fat, ranging from 45% to 75% of total calories, referred to as high-fat diets (HFDs), and derived from saturated fatty acids, polysaturated fatty acids and various combinations thereof, have been engineered to induce obesity in mice [77,78]. The source of fatty acids may vary from one to another supplier, making it difficult to standardize the diet from lab to lab [79]. Nevertheless, mice fed a “conventional” high-fat diet (i.e., with fat accounting for 45% of total calories) develop obesity, glucose intolerance, insulin resistance and hepatic steatosis, all key features of obesity and associated metabolic syndrome in humans [77,80,81]. While 3 months of HFD feeding is not sufficient to induce inflammation and fibrosis in mouse livers, a longer period of feeding, at least 6 months, may promote the development of these features [82]. Importantly, inconsistency in the development of NAFLD has been reported depending on the genetic background of the mouse strains [83]. The C57BL/6 strain seems to be more sensitive to HFD than the BALB/c strain, which exhibits a reduced hepatic lipid uptake and develops less inflammation than other strains [84]. Furthermore, C57BL/6J mice from the Jackson Laboratories, but not those from the National Institute of Health, Taconic Labs or Charles River, carry a spontaneous mutation in the *Nnt* (nicotinamide nucleotide transhydrogenase) gene, which is involved in mitochondrial redox functions contributing to NAFLD [85]. Gender differences in obesity-induced metabolic syndrome have also been reported in mice [86]. In summary, although widely used, HFD is not the best option to study NAFLD due to variations related to dietary compositions (source and nature of fatty acids), mouse strains, gender and duration of feeding. For all these reasons, during the past few years, other feeding regimens have emerged tending to reduce variability and to cover the entire spectrum of NAFLD (Figure 2).

#### 4.1.2. Western-Style or Fast-Food Diet Feeding

The Western diet represents a modern dietary pattern found in industrialized countries and characterized by high intakes of fat and sugar. It is referred to as “fast food” diet, shown to be the major risk factor for the development of obesity [87]. By analogy to human nutrition, a dietary model based on the combination of both fat and fructose and sometimes cholesterol (referred to as Western-style or fast-food diet) has been developed to induce NAFLD in mice [88,89,90]. After 6 months of fast-food diet feeding, C57BL/6 mice develop obesity, insulin resistance as well as steatohepatitis [89]. These mice also have gene expression signatures of increased fibrosis, inflammation, endoplasmic reticulum stress and lipoapoptosis, all key features found in humans with NASH [89]. Furthermore, Tsuchiya et al. have shown that feeding a fat- and fructose-rich diet for up to 16 weeks leads to hepatic iron overload, a characteristic often found in humans with NAFLD [91]. This fast-food-diet-induced obesity appears to be a more robust model for human obesity in comparison with HFD, but the lack of a standardized diet interferes with reproducibility.

#### 4.1.3. Methionine- and Choline-Deficient Diet and Derivative Feeding

Methionine- and choline-deficient (MCD) diet is a classical dietary model of NASH comprising high sucrose (40%) and fat (10%) but lacking the nutrients methionine and choline, which are essential components in animal and human nutrition [92]. Choline is the precursor of phosphatidylcholine, which is essential for very low-density lipoproteins (VLDL) production [93]. VLDL is an extracellular lipoprotein complex allowing the transport of triglycerides (TG) from the liver to the adipose tissue and muscles [94]. As for methionine, it is needed for glutathione synthesis, a major anti-oxidant protein [95]. Therefore, mice fed an MCD diet rapidly develop hepatic steatosis as a consequence of increased fatty acid uptake and decreased VLDL secretion. After two weeks, the development of steatosis is followed by cell death, inflammation and pericellular fibrosis [96,97,98]. In addition, oxidative stress and a rise in cytokines and adipokines occur in mice fed an MCD diet, which contributes to hepatic damage in this dietary model [99,100]. The MCD diet is easy to obtain and use, and it induces a more severe form of steatohepatitis than other dietary models, but it also has marked limitations. Notably, the main risk factors for the development of NAFLD in humans, namely overweight and insulin resistance, are lacking, and mice under the MCD diet lose weight progressively across feeding by up to 40% in 8 weeks [101,102]. Furthermore, several studies pointed out that the responsiveness of different mouse strains to the MCD diet varies considerably [103,104]. For all these reasons, caution is needed when using this dietary model, and the additional use of diet-induced obesity (DIO) models is desirable to examine the metabolic profile of the disease.

Choline-deficient, ethionine-supplemented (CDE) diet is derived from the MCD diet. Ethionine is a non-proteinogenic amino acid, structurally derived from methionine (i.e., an ethyl group instead of a methyl group), with the property of inducing steatohepatitis in a relatively short period-of-time [105]. Ethionine also exhibits hepatocarcinogen properties (i.e., DNA methylation interference), making CDE feeding of particular interest to study steatohepatitis-related hepatocellular carcinoma (HCC) [106]. However, this dietary model is associated with weight loss and a high rate of mortality, reaching 60% after 4 months of feeding [106,107]. To limit mortality, some studies alternate the use of a CDE diet with a normal chow diet [105]. This alternative process prevents the high rate of morbidity, whereas steatosis, inflammation and hepatocarcinogenesis are maintained [105].

The choline-deficient L-amino acid-defined (CDAA) diet is a choline-deficient diet in which proteins are replaced with an equimolar mixture of L-amino acids [82,86,108]. Like the MCD diet, CDAA inhibits fatty acid oxidation in hepatocytes, and it increases lipid synthesis, oxidative stress and inflammation, resulting in liver fibrosis, but these histological changes take longer to occur (from 3 weeks for steatosis and lobular inflammation to approximately 21 weeks for moderate fibrosis and 44 weeks for HCC) [86,109,110]. Mice fed a CDAA diet lose significantly less weight than mice under MCD or CDE diets but still do not display hepatic insulin resistance, weight gain or changes in peripheral insulin sensitivity [86,111,112]. Nevertheless, as reported by Miura et al., insulin resistance may develop upon CDAA feeding [113]. It might depend on the dietary compositions (e.g., percentage of fat) and the duration of the feeding. Hence, due to low reproducibility between the different sources of CDAA, this diet should not be used either to examine the metabolic profile of the disease.

#### 4.1.4. STAM Model

The STAM model associates a single injection of streptozotocin (STZ) as a first hit and an HFD diet as a second hit. STZ is an antibiotic produced by *Streptomyces achromogens* that acts as a DNA alkylating agent, initially used as a type I diabetes inducer due to severe pancreatic islet inflammation and destruction [114]. This model results in steatohepatitis at 8 weeks and liver fibrosis at 12 weeks and leads to HCC in virtually 100% of male mice at 20 weeks [109,115]. The hepatic lipidomic profile of STAM mice was found to be very similar to that of humans with NASH despite the chemical intervention and the absence of obesity in this model [116]. 

### 4.2. Genetic Models of NAFLD

Here, we only review genetic models that most closely replicate the disease spectrum of NAFLD, including obesity and metabolic syndrome. Other genetic mouse models of NAFLD have been reviewed elsewhere [86,117].

#### 4.2.1. *ob/ob* Mice

Genetically modified mice based on leptin deficiency have been developed to better understand NAFLD. Leptin, also named the “satiety hormone”, is a peptide secreted predominantly by the white adipose tissue to negatively regulate food intake and to increase energy expenditure [118]. Its anorexic effect is mediated by its hypothalamic receptor, which is responsible for the transmission of the satiety signal [119]. *ob/ob* mice harbor a homozygous point mutation for the gene encoding leptin. They are hyperphagic and inactive and develop severe obesity, hyperlipidemia, hyperglycemia, hyperinsulinemia and insulin resistance [120]. Although hyperphagia contributes to obesity, leptin deficiency is not an important contributor to NAFLD [121]. Indeed, serum leptin levels are normal or elevated in NAFLD [122,123,124]. In *ob/ob* mice, fat accumulation in the liver induces steatosis and hepato-lipotoxicity but rarely progress to steatohepatitis and fibrosis [125]. Hence, a second hit is needed to trigger fibrosis, such as hepatotoxic agents (e.g., CCl_4_) or MCD diet, and steatohepatitis can be studied using high-caloric diets in these animals [126,127]. 

#### 4.2.2. *db/db* Mice

In opposition to *ob/ob* mice, *db/db* mice carry a spontaneous mutation in the gene encoding the leptin receptor. Although these mice have increased levels of leptin, they develop leptin resistance conferred by the mutation of its receptor. In general, the liver histology is quite similar to that in *ob/ob* mice, and *db/db* mice are also obese, hyperphagic, insulin-resistant, hyperglycemic and hyperinsulinemic and develop hepatic steatosis [109]. However, like *ob/ob* mice, *db/db* mice do not display the whole spectrum of human NASH histopathology, and secondary stimuli are necessary to induce steatohepatitis and fibrosis [128]. Thus, the observations derived from monogenic models such as *db/db* and *ob/ob* mice may differ from the human population in which obesity is known to be a multifactorial disease. 

#### 4.2.3. The Mc4r-Deficient Mice

The binding of leptin on pro-opiomelanocortin (POMC) neurons leads to alpha-melanocyte-stimulating hormone (α-MSH) secretion [129]. This hormone mediates the anorectic signal after binding on the hypothalamic Melanocortin-4 receptor (MC4R) [129,130]. In human pathology, MC4R deficiency is responsible for 6% of monogenic obesity [131,132]. Hence, genetically modified mice with *Mc4r* deficiency have been developed to better understand NAFLD. MC4R-deficient mice are characterized by early onset of obesity associated with hyperphagia, hyperinsulinemia and hyperglycemia under a regular chow diet [133,134]. Moreover, when fed an HFD, MC4R-deficient mice develop steatohepatitis and fibrosis, and the incidence of HCC is increased after a long period of feeding [135]. Thus, MC4R-deficient mice appear suitable to examine the effects of drugs on the development of steatohepatitis when fed an HFD.

#### 4.2.4. The Srebp1c-Overexpressing Mice

*Sterol regulatory element-binding proteins* (SREBPs) are a family of transcription factors that upregulate genes involved in FFA and cholesterol synthesis [136,137]. For instance, the overexpression of SREBP1c in hepatocytes results in liver triglyceride accumulation and ER stress induction [138,139]. Moreover, these mice have increased serum FFA and triglycerides that correlate with increased visceral adipose tissue, demonstrating that pathological dysfunctions of the liver contribute to visceral adipogenicity [136,140,141,142]. However, hepatic SREBP1c overexpression is not sufficient to induce inflammation and fibrosis [141]. Hence, fast food diet feeding is secondarily needed to trigger steatohepatitis and fibrosis in these mice [143,144].

#### 4.2.5. The FATZO Mouse Model

The FATZO mouse model, also known as the MS-NASH model, was developed by crossing C57BL/6J and AKR/J mice, two strains that have a strong propensity to develop obesity when fed a high-fat diet, followed by selective inbreeding [145,146]. The crossing of these two strains and the selective inbreeding of the subsequent generations resulted in obesity, metabolic syndrome and insulin resistance predisposition. Sun et al. have shown that FATZO mice fed a fast-food diet supplemented with 5% of fructose display evidence of NASH, including hepatic steatosis, lobular inflammation, ballooning and fibrosis. The FATZO mice also had hypercholesterolemia and progressive elevation of ALT and AST compared with FATZO mice fed a normal chow diet [147]. Unlike monogenic leptin-deficient *ob/ob* and *db/db* mouse models, the FATZO mouse model is a polygenic inheritance of predisposition to obesity and diabetes, with an intact leptin pathway, thereby making it more translatable to the human disease [147]. However, selective inbreeding can contribute to a significant decrease in the genetic variability that may introduce a bias in preclinical drug testing.

Taken together, as compared with DIO, genetic models show a generally more severe disease phenotype within a shorter time frame when a diet is used to induce steatohepatitis and fibrosis. However, genetic mutations associated with these models do not reflect NAFLD etiology in humans, which limits interpretation of the results. 

## 5. Other Animal Models of Non-Alcoholic Fatty Liver Disease

### 5.1. Rat Models

Most of the dietary mouse models of NAFLD, including DIO such as HFD and Western diet, but also non-obesogenic diets such as MCD and CDAA, have been widely used in rats [148,149,150]. Rats are typically more susceptible to diet-induced NAFLD than mice, and they often progress spontaneously towards steatohepatitis and fibrosis [86,151,152]. Gender and genetic background of the different rat strains may explain their susceptibility to developing diet-induced features of NAFLD [153]. Similar to *db/db* mice, Zucker fatty (*fa/fa*) rats have a mutated leptin receptor that decreases their affinity for leptin [142,154]. These rats develop severe obesity that leads to insulin resistance and hepatic steatosis [142,155]. Like in *db/db* mice, the spontaneous progression of steatosis towards steatohepatitis is rare, and a second hit, such as a DIO, is needed [155,156]. Globally, the same variabilities seen in mice apply to the rat model of NAFLD, and dietary compositions (source and nature of fatty acids), strains, gender and duration of feeding have to be rigorously defined before experiments.

### 5.2. Non-Rodent Models

Different minipig species such as Ossabaw and Göttingen have been studied to model NAFLD [157,158]. Due to a longer lifetime than rodents, minipigs develop obesity and metabolic syndrome that is are similar to humans, which allow long-term studies [157,159]. Therefore, minipigs drug toxicity studies appear more manageable and predictive to humans than rodents [160]. However, DIO does not lead to hepatic steatosis in minipigs, most likely because of the absence of liver de novo lipogenesis [161,162]. Therefore, alternative non-obesogenic feedings have to be used, and for instance, Pedersen et al. have shown that steatohepatitis can be induced within 8 weeks in minipigs fed a CDAA diet [157]. Although minipig physiology is closer to humans than rodents, liver metabolic pathways are somehow divergent, which could limit the interpretation of the results. Furthermore, cost-effectiveness ratio and ethical and legal concerns limit the use of minipigs. 

Primates, especially rhesus monkeys, have been proposed to study NAFLD because of their genetic proximity with humans [163]. However, for ethical reasons and due to the high cost of studies, they are rarely used.

## 6. Conclusions

This review has explored the different in vivo and in vitro models used to study the pathophysiology of human NAFLD. Recent technological advances in cell culture methods (i.e., organoids, printing on a chip) promise to reduce the use of animal models for toxicological studies. Indeed, liver-on-a-chip may closely reproduce the lobular architecture of the liver, and therefore appears to be efficient to study cell-cell interactions that are essential to understand the development of NAFLD. Although the complexity of in vitro models, which better mimic cell interactions of a native liver, has dramatically increased in the past few years, these cell culture models remain imperfect and need further technological improvements and reduced cost. Therefore, in vivo models, notably murine models, are still widely used to identify metabolic pathways that lead to NASH and to investigate new therapeutics targets. Nevertheless, the extension of preclinical findings to humans are quite disappointing in many cases. This interspecies difference, which is now a challenge for research in NAFLD, could be explained for example by the difference in microbiome composition, which seems to play an important role in NAFLD pathogenesis [164]. Disease course is also different between rodents and humans, especially regarding fibrosis progression. Thus, combining NAFLD in vitro and in vivo models could be a solution to accumulate sufficient preclinical evidences to consider a clinical development of a drug.
